# Supplementary material for: Transcriptome Analysis Reveals Vimentin-Induced Disruption of Cell–Cell Associations Augments Breast Cancer Cell Migration
Source: Cells. 2022 Dec 13;11(24):4035. doi: 10.3390/cells11244035 (PMC9776984; doi:10.3390/cells11244035)
Supplement: Supplementary file 1 [file cells-11-04035-s001.zip › DEGs and enrichment analysis.zip!/DEGs and enrichment analysis/WT_VIMvsVector KEGG/UP/WT_VIMvsVector_UP.pathway_map.zip!/WT_VIMvsVector_UP.pathway_map/src/hsa05203.html]

hsa05203


- hsa:1388

- Up regulated genes

novel.600(2.78845209317737)

- hsa:1960

- Up regulated genes

ENSG00000179388(2.78490232970451)

- hsa:1388

- Up regulated genes

novel.600(2.78845209317737)

- hsa:890

- Up regulated genes

ENSG00000145386(1.72013468925064)

- hsa:890

- Up regulated genes

ENSG00000145386(1.72013468925064)

- hsa:991

- Up regulated genes

ENSG00000117399(2.41242866521263)

- hsa:8970

- Up regulated genes

ENSG00000124635(1.92753744639879)

Close
